# The experience of patient dropout from eating disorders treatment: a systematic review and qualitative synthesis

**DOI:** 10.1192/bjo.2021.792

**Published:** 2021-06-18

**Authors:** Cecilia Vinchenzo, Catherine McCombie, Vanessa Lawrence

**Affiliations:** 1 IoPNN King's College London, Lancaster University; 2 IoPPN King's College London

## Abstract

**Aims:**

Eating disorders are serious and debilitating mental health conditions associated with high relapse and mortality rates and significant psychiatric comorbidities. Research suggests approximately 50% of patients with an eating disorder dropout prematurely from treatment services, fostering poorer health outcomes and impacting significantly on patients, their families, health services and research quality. The aim of this review is to synthesise the current qualitative literature available on the patient experience of dropout from eating disorder services and understand the reasons motivating early treatment termination.

**Method:**

A systematic search was carried out and articles selected from MEDLINE, PsycINFO, EMBASE and CINAHL. Studies were included if they explored eating disorder treatment dropout using qualitative data collection or analysis methods. Study quality was critically appraised using the Critical Appraisal Skills Programme qualitative research evaluation tool. Thematic synthesis was used to interpret and synthesise themes from the primary studies.

**Result:**

Ten studies met the inclusion criteria for the systematic review. Five studies were scored as high quality and five as medium quality. 13 descriptive sub-themes encompassing the dropout experience were identified under four overarching analytical themes: inner conflict, connection and communication with others, experience of the treatment service, and factors related to progress in treatment.

**Conclusion:**

The decision to drop out from eating disorder treatment is a complex, multi-faceted issue, involving an interplay between individual, social and service-level factors. This review highlights the need for further high quality qualitative investigation into dropout experiences, with an effort to increase representation across ethnic groups and gender identities. This review also identifies the need for a reconsideration of current clinical practice and services provision with an emphasis on the use of patient perspectives to guide decision making in eating disorder services delivery and research. Moreover, the findings emphasise the need for standardised dropout definitions, fostering a unified literature base.

